# Aqueous extract of *Phragmitis rhizoma* ameliorates myelotoxicity of docetaxel in vitro and in vivo

**DOI:** 10.1186/s12906-017-1890-1

**Published:** 2017-08-09

**Authors:** Jinhee Kim, You Jin Lee, Young Ah Kim, Eun-Sang Cho, Eunna Huh, Ok-Sun Bang, No Soo Kim

**Affiliations:** 1 KM-Convergence Research Division, Korea Institute of Oriental Medicine, 1672 Yuseong-daero, Yuseong-gu, Daejeon, 34054 Republic of Korea; 2 Present address: Pathology Department, Inhalation Toxicity Research Center, Chemicals Research Bureau, Occupational Safety and Health Research Institute, Korea Occupational Safety and Health Agency, Daejeon, Republic of Korea; 3 Present address: Department of Pharmacology, Baylor College of Medicine, Houston, TX USA

**Keywords:** *Phragmitis rhizoma*, Chemotherapy, Docetaxel, Myelotoxicity, Adverse side effects

## Abstract

**Background:**

A variety of anticancer chemotherapeutics induce adverse side effects including myelotoxicity. Dried roots of *Phragmites communis* Trinius, *Phragmitis rhizoma*, have been clinically used in traditional folk medicine to relieve various symptoms like fever. In this study, we evaluated the protective effect of the aqueous extract of *Phragmitis rhizoma* (EPR) against docetaxel-induced myelotoxicity in vitro and in vivo.

**Methods:**

The in vitro myelo-protective effect of EPR was evaluated using the colony forming unit (CFU) assay with hematopoietic progenitor cells. The in vivo efficacy of EPR was evaluated in myelosuppressed C57BL/6 male mice which were induced by repeated intraperitoneal injections of 30 mg/kg docetaxel for 3 times. EPR was orally administered for 4 days to docetaxel-induced myelosuppressed C57BL/6 male mice which were induced by intraperitoneal injection of 30 mg/kg docetaxel for 3 times: Group 1 (vehicle control, *n* = 10), Group 2 (docetaxel plus vehicle, *n* = 10), Group 3 (docetaxel plus EPR 30 mg/kg, *n* = 10), Group 4 (docetaxel plus EPR 100 mg/kg, *n* = 10) and Group 5 (docetaxel plus EPR 300 mg/kg, *n* = 10). Whole blood counts were measured automatically, and immune organs were histologically examined. Expression of immunomodulatory cytokines was measured by quantitative real-time polymerase chain reaction or enzyme-linked immunosorbent assay. The toxicity of EPR itself was evaluated in normal human cell lines including IMR-90, foreskin fibroblast and human umbilical vein endothelial cells. The hepatotoxicity of EPR was predicted by multi-parametric assays involving cell viability, caspase 3/7 activity, GSH contents and LDH leakage using the HepaRG hepatic cell line.

**Results:**

Co-treatment of EPR or its major component, *p*-hydroxycinnamic acid, increased the numbers of hematopoietic CFU counts in the docetaxel-induced in vitro myelotoxicity assay system. The in vitro protective effect of EPR against docetaxel toxicity was replicated in a myelosuppressed animal model: white blood cells, neutrophils, lymphocytes and red blood cells rebounded; bone marrow niche and structural integrity of the thymus were preserved; and the expression of immune-stimulating cytokines including IL3, IL6, SCF and GM-CSF was enhanced. Furthermore, EPR and *p*-hydroxycinnamic acid promoted the proliferation of primary splenocytes and thymocytes. In the toxicity assays, no remarkable signs related with toxicity were observed in all tested normal human cells and HepaRG.

**Conclusions:**

EPR has the potential to ameliorate docetaxel-mediated myelotoxicity in both in vitro and in vivo models. However, the identification of the responsible active components and the precise underlying myelo-protective mechanism of EPR need to be elucidated before novel drug development using EPR can precede.

**Electronic supplementary material:**

The online version of this article (doi:10.1186/s12906-017-1890-1) contains supplementary material, which is available to authorized users.

## Background

Cancer is one of the leading causes of deaths worldwide [1]. Chemotherapy is a standard treatment of cancers along with surgical operation and radiotherapy. Cytotoxic chemotherapy generally targets uncontrolled growth of cancer cells, and therefore, fast growing normal bystander cells like bone marrow cells are also affected by these therapies. As a member of taxane anticancer agents, docetaxel, brand name Taxotere, is widely used to treat a wide range of solid tumors including breast, ovary, prostate and non-small-cell lung cancers [2]. It suppresses tumor cell growth by inhibiting mitotic spindle assembly leading to G2/M cell cycle arrest and eventually apoptotic cell death [2, 3]. However, the limitation of docetaxel in clinical use is its side effects, such as hair loss, numbness, vomiting and especially myelosuppression. Serious hematopoietic injuries like decreased bone marrow cells lead to a delay in the drug treatment schedule, dose reduction, or early termination of the chemotherapy. Therefore, suitable treatment for myelosuppression is very important for the overall outcome of the chemotherapy as well as for the quality of life of cancer patients.

Traditional medicines have emerged as alternative treatments for chemotherapy-induced myelosuppression. Recent studies involving animal models or human trials have shown that traditional medicine, especially medicinal herb extracts, have benefits in the treatment of chemotherapy-induced myelosuppression. For example, *Fufang E’jiao Jiang*, a famous formulation used in traditional Chinese medicine (TCM), efficiently relieved radiotherapy/chemotherapy-induced myelotoxicity in animal models [4]. *Panax ginseng* with the active ingredient ginsenoside was able to recover experimental animals from 5-fluorouracil-induced myelotoxicity [5]. Prescription of TCM decoctions, *Jian Pi* and *Yi Qi Yang Xue Sheng Sui*, could lower the risk of leukopenia and neutropenia and febrile neutropenia in breast cancer patients receiving anthracyclines combined with paclitaxel or docetaxel [6]. Thus, traditional herbal medicines are considered good starting natural sources for the development of novel pharmaceuticals that would manage chemotherapy-induced myelosuppression.

*Phragmites communis* Trinius is a perennial plant that is globally distributed in the temperate and boreal regions of the world [7]. The dried root of *P. communis*, *Phragmitis rhizoma*, has been prescribed in traditional Korean medicine (TKM) to relieve fever and vomiting and to nourish the body fluid (Korean Traditional Knowledge Portal) [8]. It is registered in the Korean Herbal Pharmacopoeia as well as in the Chinese Pharmacopeia. In our in vitro activity screening test, the aqueous extract of *Phragmitis rhizoma* (EPR) exhibited a protective effect against myelotoxicity induced by diverse chemotherapeutic agents with different mechanisms of action. In this study we show that EPR can ameliorate chemotherapy, especially, docetaxel-induced myelotoxicity in both in vitro hematopoietic colony forming unit (CFU) assay as well as in vivo animal models with primary endpoints focusing on changes in blood cell counts and microenvironmental structures of immune organs. To the best of our knowledge, this is the first report showing the pharmaceutical value of *Phragmitis rhizoma* to treat chemotherapy-induced myelosuppression in cancer patients.

## Methods

### Chemicals and reagents

*p*-hydroxycinnamic acid, a reference standard for chromatographic analysis, was purchased from ChemFaces (Wuhan, Hubei, China). The purity of the *p*-hydroxycinnamic acid was ≥99.1% for high-performance liquid chromatography (HPLC). HPLC grad acetonitrile and analytical grad formic acid were purchased from JT Baker chemicals (Center Valley, PA, USA) and Wako chemicals (Osaka, Japan), respectively. Deionized distilled ultra-pure water was produced by Millipore RiQs & Milli-Q-Gradient water purification system (Millipore, Bedford, MA, USA). Docetaxel was purchased from LC laboratories (D1000, Woburn, MA, USA). CPT11 (camptothecin, C9911) and doxorubicin (D1515) were obtained from Sigma (St. Louis, MO, USA).

### Plant materials and extract preparation

*Phragmitis rhizoma* was purchased from Kwangmyungdang Medicinal Herbs Co. (Ulsan, Republic of Korea) and identified by Dr. Goya Choi, K-herb Research Center, Korea Institute of Oriental Medicine, Republic of Korea. A voucher specimen (KIOM-CRC-#172) was deposited at the KM Convergence Research Division, Korea Institute of Oriental Medicine. The dried *Phragmitis rhizoma* (500.0 g) was finely ground using a mechanical pulverizer and then subjected to repeated extraction in boiling distilled water (100 °C, 2 h, 10 L × 2 times,) using a reflux extraction system from KOC Biotech (Daejeon, Republic of Korea). The extract solution was filtered through cotton wool, concentrated in the EYELA rotary evaporator system (Tokyo Rikakikai, Tokyo, Japan) and freeze-dried (FD8518, Ilshin Biobase, Dongducheon, Republic of Korea) to produce a final water extract (EPR, 60.21 g, 12.0%). The extract was stored at 4 °C in a dark plastic bottle with a silica gel desiccant until use.

### Ultra-high performance liquid chromatographic (UHPLC) analysis of the extract

Phytochemical analysis of the EPR was performed with the 1290 Infinity UHPLC system (Agilent Technologies, Waldbronn, Germany). The EPR was dissolved in 50% (*v*/v) methanol at 10 mg/ml. A reference standard stock solution was prepared by dissolving *p*-hydroxycinnamic acid in 50% methanol at 1 mg/ml. The EPR and standard stock solution were cleared with a 0.22 μm syringe filter. The standard stock solution was further 10-fold diluted with 50% methanol right before the UHPLC analysis. The EPR or diluted standard solution was loaded on the auto-sampler which was maintained at 4 °C. Chromatographic analysis was performed on the ZORBAX Eclipse Plus C_18_ column (2.1 × 50 mm, 1.8 μm pore size, Agilent Technologies, Santa Clara, CA, USA). The linear gradient mobile phase was 0.1% formic acid in water (A) and 100% acetonitrile (B). The detailed conditions for the UHPLC analysis are summarized in Table 1. Chromatographic data were further processed by the OpenLAB CDS (ChemStation Edition) software (Agilent).

**Table 1:** UHPLC analytical conditions

Parameter		Chromatographic parameters	
Column temperature		40 °C	
Detection wave length		UV at 290 nm	
Injection volume		2.0 μl	
Flow rate		0.3 ml/min	
Run time		60 min	
Post time		10 min	
Mobile phase	Time (min)	A (0.1% formic acid)	B (acetonitrile)
	0	100	0
	50	60	40
	51	0	100
	60	0	100

### In vitro toxicity assays

Normal human lung fibroblast IMR-90 (CCL-186) and neonatal foreskin fibroblast (HFFn, PC501A-HFF) cells were obtained from the American Type Culture Collection (Rockville, MD, USA) and Systems Biosciences (Mountain View, CA, USA), respectively. They were maintained in DMEM basal media which included 10% (*v*/v) heat inactivated fetal bovine serum (FBS), 100 U/ml penicillin and 100 μg/ml streptomycin antibiotics. DMEM, FBS and antibiotics were obtained from Thermo Fisher Scientific (Waltham, MA, USA). Human umbilical vein endothelial cells (HUVEC) were purchased from Lonza (Walkersvill, MD, USA) and maintained in EGM-2 basal medium containing endothelial growth medium supplements (Lonza). To determine the toxicity of the EPR itself, cells were inoculated at 50,000 cells/well in a 96-well plate and incubated for 24 h at 37°C in a humidified atmosphere of 5% CO_2_. Then, the cells were exposed to various concentrations of EPR for 48 h. Cell viabilities were determined using the Ez-Cytox cell viability assay kit (Daeil Lab Service, Seoul, Republic of Korea) according to the manufacturer’s instructions.

The hepatotoxicity of the EPR was assayed in vitro with the human hepatic cell line HepaRG. Frozen cells, William’s E basal medium, supplements for general maintenance or the toxicity assay were purchased from Thermo Fisher Scientific. Preparation of the media for general maintenance or the toxicity assay and differentiated HepaRG were done according to the manufacturer’s instructions. Cells that were differentiated in a collagen type I pre-coated 96-well plate (Corning, Kennebunk, ME, USA) were exposed to various concentrations of EPR for 24 h and then, hepatotoxicity parameters including cell viability (WST-1 reagent, Roche, Mannheim, Germany), intracellular glutathione (GSH) contents (GSH-Glo glutathione assay system, Promega, Madison, WI, USA), caspase 3/7 activity (Caspase-Glo 3/7 assay system, Promega) and lactate dehydrogenase (LDH) leakage (Cytotoxicity Detection kit, Roche) were determined as described by the manufacturer’s instructions.

### CFU assay of hematopoietic progenitor cells

The in vitro CFU assay has been used to evaluate drug-related myelotoxicity in previous studies [9, 10] and the CFU-GM assay was endorsed by the members of European Centre for the Validation of Alternative Methods (ECVAM) Scientific Advisory Committee (ESAC) as a substitute to experimental animals to predict myelotoxicity in humans [11]. Mouse bone marrow nucleated cells (BMNCs) were isolated from the femoral bones of C57BL/6 mice as described previously with slight modifications [12]. In brief, femoral bones were removed from a CO_2_ euthanized mouse under sterile conditions and immersed in ice-cold Hank’s balanced salt solution (HBSS, Thermo Fisher Scientific) containing 100 U/ml penicillin and 100 μg/ml streptomycin. Both epiphyses of the femurs were removed with sterile scissors, and bone marrow cells were collected by strongly flushing the diaphysis with ice-cold HBSS using a 1 ml syringe. Red blood cells (RBC) were lysed using a 0.9% (*w*/*v*) NH_4_Cl solution (StemCell Technologies, Cambridge, MA, USA). After washing with HBSS, BMNCs were resuspended in DMEM containing 1% FBS, 100 U/ml penicillin and 100 μg/ml streptomycin. Viable cells were counted using the ADAM-MC auto cell counter (NanoEnTek, Seoul, Republic of Korea). Viable 1×10^4^ BMNCs were homogeneously dispersed in the Methocult GF M3434 medium (StemCell Technologies) containing a combination of EPR and anticancer drugs, plated in a 24-well culture plate (StemCell Technologies) and incubated at 37°C in a humidified atmosphere of 5% CO_2_. After 7 days, colonies consisting of more than 30 cells were manually counted under an inverted microscope (IX71, Olympus, Center Valley, PA, USA).

### Experimental animals

Six-week-old C57BL/6 male mice were supplied by OrientBio (Seongnam, Republic of Korea) and housed in a specific pathogen free laboratory animal care facility. Microbiological status of animals was evaluated and documented by the supplier. All animals were housed (5 mice/polycarbonate cage) and acclimated for 1 week before experiments in an environmentally controlled vivarium (temperature, 22 ± 2 °C; humidity, 45 ± 10; and dark and light cycle, 12 h/12 h). The animals accessed to water and commercial standard chow ad libitum. All procedures for the animal studies were in accordance with the relevant national raw and reviewed by the Institutional Animal Care and Use Committee of Korea Institute of Oriental Medicine (Protocol #16–075).

### Animal studies

Docetaxel was dissolved at 60 mg/ml in a 1:1 mixture of ethanol and polysorbate 80 (Sigma) to make a stock solution. They were always freshly prepared when needed. To minimize crystallization of the docetaxel, the stock solution was further diluted to 2.4 mg/ml with injectable normal saline (Samyang Anipham, Seoul, Republic of Korea) right before injection. Experimental animals were randomly allocated in to 5 groups. Animals (Group 2 to Group 5) received docetaxel intraperitoneally at 30 mg/kg/day or vehicle (Group 1) for 3 consecutive days. Then, mice orally received EPR (Group 3, 30 mg/kg; Group 4, 100 mg/kg; Group 5, 300 mg/kg; *n* = 10 mice/group) or 0.5% (*w*/*v*) carboxymethylcellulose (CMC, Group 2, *n* = 10 mice, Sigma, St. Louis, MO, USA) as a vehicle for 4 days. At day 5, the animals were anaesthetized by intraperitoneal injection of 50 mg/kg pentobarbital solution (Entobar, Hanlim Pharmaceuticals Inc., Seoul, Republic of Korea). Whole blood was collected from the heart using a 1 ml syringe with 25 gauge pre-coated with 10% (*w*/*v*) ethylenediaminetetraacetic acid (EDTA) dipotassium salt (Sigma) for the blood cell counts. Half of the spleens were rapidly frozen in liquid nitrogen and store at −70 °C until total RNA preparation. The other half of the spleen and the whole thymus and femurs isolated from the mice were soaked and fixed in a neutral buffered formalin (pH 6.8–7.2, BBC Biochemical, Mount Vernon, WA, USA) for 72 h at 4 °C with gentle shaking.

### Complete blood count (CBC) analysis

CBC analysis of EDTA-treated whole blood was determined using a HEMAVET 950 automatic analyzer (Drew Scientific, Miami Lakes, FL, USA).

### Isolation and culture of splenocytes and thymocytes

All procedures except for animal euthanasia were performed in an aseptic area. Thymus and spleen were isolated from a normal 6-week-old male C57BL/6 mouse that was euthanized by CO_2_ inhalation. The tissues were transferred to a 70 μm pore-sized meshed cell strainer (Thermo Fisher Scientific) that was submerged in ice-cold RPMI medium containing 100 U/ml penicillin and 100 μg/ml streptomycin. The tissues were gently mashed with a 1 ml syringe rubber plunger, and then, the cells were transferred to a 50 ml conical tube. The cell were washed once using RPMI with antibiotics and collected at 1600 rpm for 5 min at 4 °C. Then, the cells were subjected to osmotic shock by adding 450 μl of sterile distilled water for 5 s to lyse the RBC and neutralized by adding 50 μl of 10× phosphate buffered-saline (PBS, pH 7.4). The cells were centrifuged and resuspended in a 1 ml pre-warmed complete medium which was RPMI supplemented with 10% FBS and antibiotics. Viable cells at 5 × 10^5^ in 95 μl of complete medium were seeded in a 96-well culture plate (Nunc, Naperville, IL, USA) pre-loaded with 5 μl of 20× concentrated drugs and incubated at 37 °C in a humidified 5% CO_2_ incubator. After 48 h, cell growth was determined using a WST-1 cell proliferation reagent (Sigma) according to the manufacturer’s instruction.

### Enzyme-linked immunosorbent assay

Quantification immunomodulatory cytokines released from splenocytes was determined using commercially available enzyme-linked immunosorbent assay (ELISA). The ELISA kits detecting murine interleukin-3 (IL3, ab113345) and IL6 (ab100712) were obtained from Abcam (Cambridge, MA, USA). The kits for murine interferon-gamma (IFNγ, MIF00) and tumor necrosis factor-alpha (TNFα, MTA00B) were obtained from R&D systems (Minneapolis, MN, USA). The culture medium was clarified by centrifugation at 12,000 rpm for 1 min, and the supernatant was subjected to ELISA assay following the manufacturer’s instruction. Horseradish peroxidase-mediated 3,3',5,5'-tetramethylbenzidine color development was quantified at 450 nm using the Emax microplate reader (Molecular Devices, Sunnyvale, CA, USA). The concentrations of the cytokines released in the medium were quantified by comparison with serially diluted standards that were included in the kits.

### Determination of cytokine expression in splenic tissues and BMNCs

Total RNAs were isolated from frozen spleens using the Easy-Spin™ total RNA extraction kit (iNtRON Biotechnology, Seoul, Republic of Korea) according to the manufacturer’s instructions. BMNCs were prepared as described above. After determining the cell viability, viable cells at 4-5 × 10^6^ in 490 μl of RPMI supplemented with 10% FBS and antibiotics were plated in a 12-well culture plate (Nunc) pre-loaded with 10 μl of 50× concentrated drugs. After 24 h, the cells were washed with ice-cold PBS and total RNAs were isolated using the Easy-Spin™ total RNA extraction kit. Single-stranded cDNA was synthesized from 1 μg of total RNA using the iScript™ cDNA synthesis kit (Bio-Rad, Hercules, CA, USA). Quantitative real-time polymerase chain reaction (qPCR) was performed using the SsoAdvanced Universal SYBR Green Super mix (Bio-Rad), and fluorescence intensities were detected by the CFX96 real time system (Bio-Rad). All primers except those for IL3 (MQP029461, GeneCopoeia, Rockville, MD, USA) were synthesized and provided by Genotech (Daejeon, Republic of Korea). The information for the primer sets and enzyme reactions are summarized in Table 2.

**Table 2:** Primers and quantitative PCR conditions to determine expression of murine immunomodulatory cytokines

Target	Forward (5**′ →** 3′)	Reverse (5′ → 3′)	GenBank Accession #
GAPDH	TTGATGGCAACAATCTCCAC	CGTCCCGTAGACAAAATGGT	NM_001001303
GM-CSF	CCGTAGACCCTGCTCGAATA	TGCCTGTCACATTGAATGAA	NM_009969.4
IFNγ	ATGAACGCTACACACTGCATC	CCATCCTTTTGCCAGTTCCTC	NM_008337
IL1β	GCAACTGTTCCTGAACTCAACT	ATCTTTTGGGGTCCGTCAACT	NM_008361
IL3	All-in-One™ qPCR primer (MQP029461)		NM_010556.4
IL6	TAGTCCTTCCTACCCCAATTTCC	TTGGTCCTTAGCCACTCCTTC	NM_031168
IL7	TTCCTCCACTGATCCTTGTTCT	AGCAGCTTCCTTTGTATCATCAC	X07962.1
IL12β	TGGTTTGCCATCGTTTTGCTG	ACAGGTGAGGTTCACTGTTTCT	NM_008352
SCF	ATGTTCCCCGCTCTCTTTGG	GTGTGGCATAAGGGCTCACT	NM_013598.2
TNFα	CCCTCACACTCAGATCATCTTCT	GCTACGACGTGGGCTACAG	NM_013693
Step	Temperature (°C)	Time (s)	# of cycle
Activation	95	30	1
Amplification	95	15	40
	60	30	
Melting-curve	65–95	2 s/step	1

### Histological analysis

Formalin fixed femurs were treated with Decalcifying solution-Lite (Sigma) according to the manufacturer’s instructions. Formalin-fixed spleen, thymus and decalcified femurs were embedded in paraffin. Microscopic slides with a 4 μm thickness were prepared from the paraffin-embedded tissues and then subjected to hematoxylin-eosin (H&E) staining. Histological examination and capture of digital images were performed under a light microscope (BX41, Olympus).

### Statistics

Statistical analyses were done with SigmaPlot (v11.0, Systat Software, San Jose, CA, USA). Differences among the means were compared with one-way analysis of variance followed by the Tukey *post-hoc* test. The differences with a *p* < 0.05 were considered significant.

## Results

### Chromatographic fingerprint analysis of the EPR

**Fig. 1:**
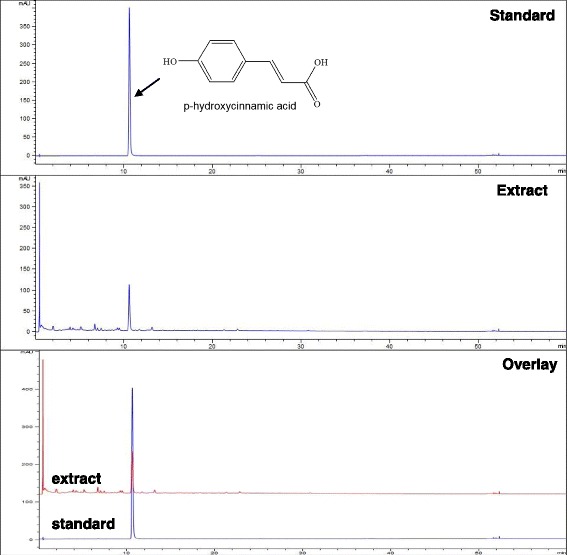
Chromatographic analysis of *Phragmitis rhizoma* extract. UHPLC was performed on the commercially available standard, *p*-hydroxycinnamic acid, and *Phragmitis rhizoma* extract. Two chromatograms were overlaid. UV detection was performed at 290 nm wavelength

The components of EPR were separated by UHPLC as described earlier. *p*-hydroxycinnamic acid was previously isolated from the *Phragmitis rhizoma* [13]. We compared the EPR and a reference standard of *p*-hydroxycinnamic acid in a parallel HPLC chromatogram. The chemical structure of the standard *p*-hydroxycinnamic acid and the representative UHPLC chromatograms of the EPR and standard are shown in Fig. 1. Phytochemical analysis of the EPR revealed that *p*-hydroxycinnamic acid was detected as a major component of the EPR which was perfectly matched with the standard in the liquid chromatogram at a retention time of 10.5 min. It accounted for 36% of total UV chromatogram at 290 nm. Other polar products (far left peaks in the Fig. 1) and minor small peaks around *p*-hydroxycinnamic acid accounted for the rest of UV chromatogram.

### In vitro protective effect of the EPR and *p*-hydroxycinnamic acid against the myelotoxicity of anticancer agents

**Fig. 2:**
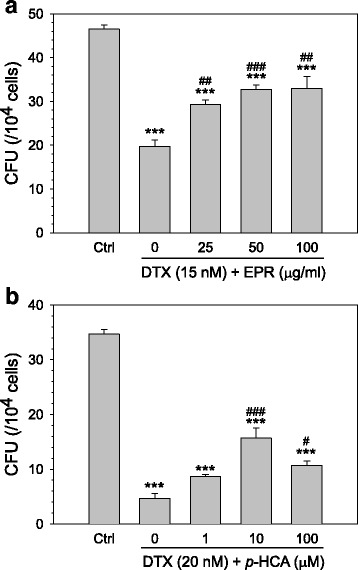
In vitro myelo-protective effect of *Phragmitis rhizoma* extract and its major component, *p*-hydroxycinnamic acid. CFU assay using mouse BMNCs was performed in the presence of combination of docetaxel (DTX) and increasing concentrations of **a** *Phragmitis rhizoma* extract (EPR, 25–100 μg/ml) or **b** *p*-hydroxycinnamic acid (*p*-HCA, 1–100 μM). The number of colonies per 10^4^ BMNCs that were inoculated on the semi-solid medium was manually counted. Data are represented as means ± SEM of 4 (EPR) or 3 (*p*-HCA) repeated experiments. ***, *p* < 0.001 vs. vehicle-treated control (Ctrl); ^#^,^##^ and ^###^, *p* < 0.05, *p* < 0.01 and *p* < 0.001 vs. docetaxel only treated group, respectively

BMNCs isolated from a normal C57BL/6 mouse were treated with myelosuppression-inducing doses of anticancer drugs. Docetaxel at 15 nM significantly reduced the hematopoietic CFU counts by 57.5% when compared to the vehicle-treated control (Fig. 2a). However, simultaneous treatment with EPR (25–100 μg/ml) increased the CFU counts in a dose-dependent manner, and the CFU counts increased up to 71% at 100 μg/ml EPR. In addition, we further evaluated the myelo-protective effect of *p*-hydroxycinnamic acid, a major component of EPR, in the presence of 20 nM docetaxel. As shown in Fig. 2b, *p*-hydroxycinnamic acid could reduce docetaxel toxicity in a dose-dependent manner and the CFU counts increased up to 50% at 10 μM *p*-hydroxycinnamic acid. The myelo-protective effect of the EPR was further evaluated against 2 other anticancer agents, CPT11 and doxorubicin, with have different anticancer mechanisms of action. For both anticancer agents, EPR efficiently protected the cells from CPT11- or doxorubicin-induced myelotoxicity in a dose-dependent manner (Additional file 1: Figure S1). The protective potential of EPR was most evident in the docetaxel model; thus, we further investigated its in vivo efficacy in a docetaxel-induced myelotoxic animal model.

### Protective effect of the EPR against docetaxel-induced myelotoxicity in vivo

The myelo-protective potential of the EPR was further evaluated using an experimental animal. Myelosuppression was induced by repeated injection of docetaxel (30 mg/kg; 3 times) and the mice received EPR for 4 days. Hematological analysis of the whole blood revealed that the blood counts of white blood cells (WBC, 39%), neutrophils (38%), lymphocytes (46%), RBC (15%) and hemoglobin concentration (12%) were decreased by docetaxel; however, co-treatment with EPR at 30–300 mg/kg recovered the mice from the myelotoxicity of the docetaxel in a dose dependent manner with 82, 88, 77, 89 and 90.8% WBC, neutrophils, lymphocytes, RBC and hemoglobin, respectively, at 300 mg/kg EPR (Table 3). Platelets were significantly increased by docetaxel treatment which was slightly attenuated by EPR.

**Table 3:** Hematological effect of EPR in docetaxel-induced myelosuppressed mice

Treatment	WBC (x10^6^/ml)	Neutrophils (x10^6^/ml)	Lymphocytes (x10^6^/ml)	RBC (x10^9^/ml)	Hemoglobin (mg/dl)	Platelets (x10^6^/ml)
Control	3.44±0.24	0.48±0.02	2.88±0.23	9.11±0.18	13.64±0.23	659±13
DTX+Vehicle	2.09±0.19***	0.30±0.01***	1.56±0.22***	7.72±0.15***	11.96±0.29***	1085±46***
DTX+EPR(30)	2.52±0.15**	0.47±0.06##	1.83±0.10***	8.04±0.13***	12.23±0.13***	992±71***
DTX+EPR(100)	2.59±0.13**,#	0.41±0.06	2.09±0.10**,#	7.88±0.13***	11.94±0.21***	999±48***
DTX+EPR(300)	2.84±0.30	0.42±0.07	2.22±0.25	8.11±0.18***	12.39±0.25**	949±59***

Data were presented as means±SEM of n=10 mice. **, p<0.01 vs. control; #, p<0.05, ##, p<0.01 vs. DTX+Vehicle treated group

### Histological effects of the EPR on docetaxel-induced myelotoxicity

**Fig. 3:**
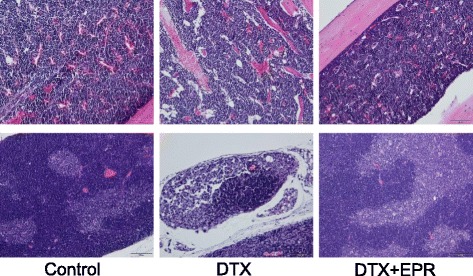
Histological evaluation of myelo-protective effect of *Phragmitis rhizoma* extract. Femoral bones (*upper*) and thymuses (*lower*) of normal control (*left*), docetaxel (DTX, 30 mg/kg × 3 times) only (*middle*) and docetaxel and *Phragmitis rhizoma* extract (EPR, 300 mg/kg) co-treated (DTX + EPR, *right*) mice were subjected to H&E staining

The bone marrows and thymus of the experimental mice that were exposed to docetaxel in the presence or absence of EPR were histologically examined (Fig. 3). When compared to the vehicle-treated control mice, decreased numbers of bone marrow cells (hypocellularity) and replacement with adipocytes in the marrow cavity were observed in the docetaxel-treated mice. On the other hand, EPR co-treatment remarkably reduced the docetaxel-induced hypocellularity of the bone marrow and the appearance of adipocytes and moderately maintained microenvironmental niches in the femoral bones. The thymic tissues of the docetaxel-treated mice were characterized by disappearance of the borderline between the cortex and medulla and by a loss of lymphocytes, which were also reduced by the EPR treatment.

### Effects of the EPR on the expression of immune-stimulating cytokines in spleens

**Fig. 4:**
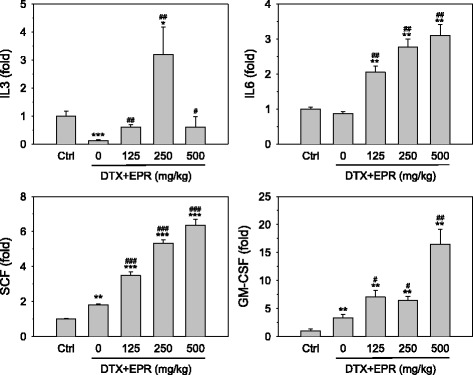
Expression of immune-stimulating cytokines from splenic tissues. The expression of cytokines were determined by qPCR using total RNA isolated from splenic tissues of mice exposed to combination of docetaxel (DTX) and *Phragmitis rhizoma* extract (EPR). Data are represented as means ± SEM of 4 repeated experiments. ** and ***, *p* < 0.01 and *p* < 0.001 vs. vehicle-treated control (Ctrl); ^#^, ^##^ and ^###^, *p* < 0.05, *p* < 0.01 and *p* < 0.001 vs. docetaxel only treated group, respectively

Expression of immune-stimulating cytokines such as IL3, IL6, stem cell factor (SCF) and granulocyte/macrophage-colony stimulating factor (GM-CSF) was examined in the spleens of mice by qPCR. As shown in Fig. 4, IL3 was significantly decreased by docetaxel, which was recovered by the EPR treatment. IL3 was increased by 250 mg/kg EPR and then returned to basal level at 500 mg/kg EPR. Treatment with docetaxel alone did not change the IL6 expression; however, it was dose-dependently increased by EPR and increased by 3-fold at 500 mg/kg EPR. SCF and GM-CSF were increased by the docetaxel treatment alone and augmented by the EPR co-treatment in a dose-dependent manners. SCF and GM-CSF were increased by 6- and 15-fold at 500 mg/kg EPR, respectively.

### Effects of the EPR on the cell proliferation of splenocytes and thymocytes

**Fig. 5:**
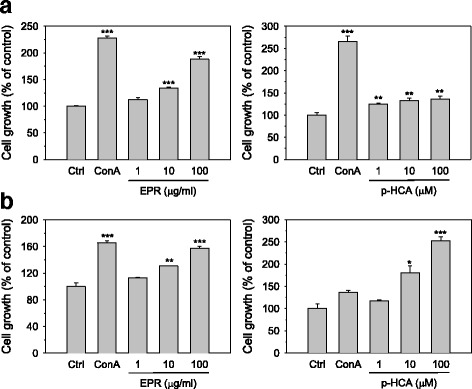
Effect of *Phragmitis rhizoma* extract on proliferation of splenocytes and thymocytes. Splenocytes (**a**) or thymocytes (**b**) were incubated in the presence of *Phragmitis rhizoma* extract (EPR, 1–100 μg/ml) or its major component of EPR, *p*-hydroxycinnamic acid (p-HCA, 1–100 μM). Concanavalin A (ConA) was included as a control mitogen. The cell proliferation was determined after 48 h of drug exposure. Data are represented as means ± SEM of triplicated (splenocytes) or duplicated (thymocytes) experiments. *, ** and ***, *p* < 0.05, *p* < 0.01 and *p* < 0.001 vs. vehicle-treated control (Ctrl)

Splenocytes and thymocytes were isolated from normal mice and then incubated in the presence of EPR or *p*-hydroxycinnamic acid. EPR and *p*-hydroxycinnamic acid promoted cell the proliferation of splenocytes in a dose dependent manner, and cell proliferation was increased by 88% and 36% at 100 μg/ml EPR and 100 μM *p*-hydroxycinnamic acid, respectively (Fig. 5). Enhanced cell proliferation by EPR and *p*-hydroxycinnamic acid was observed in the thymocytes, and cell proliferation was increased by 57% and 250% at 100 μg/ml EPR and 100 μM *p*-hydroxycinnamic acid, respectively.

### Effects of the EPR on the cytokine expression of splenocytes and bone marrow cells

**Fig. 6:**
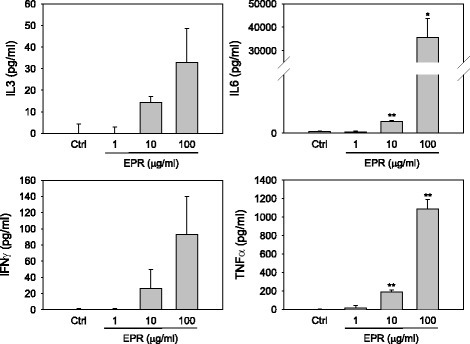
Effect of *Phragmitis rhizoma* extract on cytokine expression from normal splenocytes. The splenocytes isolated from normal mouse were exposed to increasing concentrations of *Phragmitis rhizoma* extract (EPR, 1–100 μg/ml) for 48 h and the culture media were subjected to ELISA. Data are represented as means ± SEM of duplicated experiments. * and **, *p* < 0.05 and *p* < 0.001 vs. vehicle-treated control (Ctrl)

Splenocytes isolated from a normal mouse were exposed to increasing concentrations of EPR (1–100 μg/ml) for 48 h. Cytokines released from the cells were quantified using ELISA. EPR significantly increased the expression of IL3, IL6, IFNγ and TNFα in a dose-dependent manners (Fig. 6). Among them, IL6 and TNFα were remarkably increased by 100 μg/ml EPR to approximately 35,000 and 1100 pg/ml, respectively.

Expression of immune-modulatory cytokines in bone marrow cells was determined by qPCR as described earlier. Isolated BMNCs from a normal mouse were exposed for 48 h to increasing concentrations of EPR (2–200 μg/ml), and total RNAs were subjected to qPCR analysis. EPR treatment promoted the expression of IFNγ and IL1β but inhibited IL6 in the bone marrow cells. The expression of IL7, IL12β and TNFα did not change by the EPR treatment (Table 4).

**Table 4:** Changes in cytokine expression from bone marrow cells by EPR

Cytokines	Control	Relative expression (fold)		
		EPR(2)	EPR(20)	EPR(200)
IFNγ	1.0±0.1	1.5±0.1*	5.9±0.3***	2.5±0.0***
IL1β	1.0±0.1	2.0±0.1***	2.4±0.0***	4.1±0.1***
L6	1.0±0.0	0.4±0.0***	0.3±0.0***	0.3±0.0***
IL7	1.0±0.3	0.7±0.2	1.1±0.1	1.2±0.2
IL12β	1.0±0.1	1.0±0.2	1.1±0.1	0.9±0.1
TNFα	1.0±0.0	1.0±0.0	0.8±0.1	1.0±0.3

Relative expression was determined by comparing to normal control. Data are presented as means±SEM of triplicated experiments. *, ** and **, p<0.05, p<0.01 and p<0.001 vs. vehicle-treated control

### EPR toxicity in normal cells

**Fig. 7:**
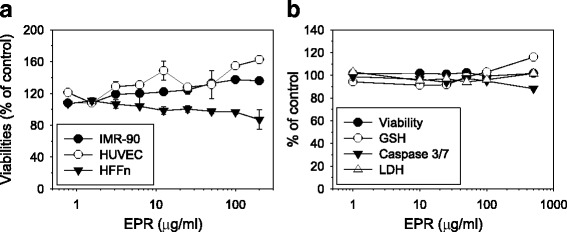
In vitro toxicity of *Phragmitis rhizoma* extract in normal cells. **a** Normal human lung fibroblast (IMR-90), neonatal foreskin fibroblast (HFFn), endothelial (HUVEC) cells were exposed to serially diluted EPR. The relative cell viabilities were determined after 48 h compared with vehicle treated cells. **b** Human hepatic cell line, HepaRG, was treated with serially diluted EPR for 24 h. Cell viability, intracellular GSH content, caspase 3/7 activity and LDH release were quantified as described earlier after 24 h drug treatment. Data are represented as means ± SEM of duplicated (**a**) or triplicated (**b**) experiments

The toxicity of the EPR was evaluated in 2 normal human fibroblast cells (IMR-90 and HFFn) and in an endothelial cell (HUVEC). The cells were exposed to EPR (0–200 μg/ml), and their relative viabilities were determined after 48 h. As shown in Fig. 7a, no remarkable loss of cell viabilities was observed in any types of cells up to 200 μg/ml EPR. They maintained viabilities over 80% when compared to the vehicle control. On the other hand, a slight increase in the cell viabilities was observed for both IMR90 and HUVEC.

The hepatotoxicity of the EPR was also inferred by measuring hepatotoxic multi-parameters in vitro using HepaRG. Cells were exposed to EPR (0–500 μg/ml) for 24 h, and then, relative changes of each parameter were determined by comparing with the vehicle treated cells. As shown in Fig. 7b, no remarkable hepatotoxic signs, such as loss of cell viability, caspase 3/7 activation, decrease of intracellular GSH contents and LDH leakage from the cells, were observed for up to 500 μg/ml EPR. The calculated toxic concentrations that decrease the response by 50% (TC_50_) for all parameters were higher than 500 μg/ml.

## Discussion

Docetaxel is widely prescribed as an adjuvant chemotherapy to treat various types of solid tumors [2]. However, the adverse side effects of docetaxel, such as myelosuppression, limit its clinical use [2]. Modification of the docetaxel formulation using nanotechnologies is under investigation to minimize the docetaxel myelotoxicity [2]. However, hematopoietic growth factors (HGFs) like recombinant granulocyte-colony stimulating factor (G-CSF) and erythropoietin are now first-line choices for the treatment of myelosuppression in cancer patients. The drawback of HGFs, however, are their high costs and their own adverse effects like osteomuscular pain, arthralgia and allergic reactions [4]. Furthermore, a recent study raised a critical safety issue of G-CSF in cancer patients, that is, its potential to promote tumor growth by enhancing neovascularization in a tumor [14]. Therefore, the development of efficient and safer therapeutics or preventives are still needed to manage cancer patients.

A variety of traditional herbal medicines have been studied to evaluate their preventive or therapeutic efficacies against chemo−/radiotherapy-induced myelosuppression, and they have shown some promising pharmaceutical values. They have consisted of extracts of a single medicinal herb [15–17], an herbal decoction or extract combination [4–6, 18–21], or active fractions or single components isolated from medicinal herbs [1, 22–25]. Their protective effects have been investigated using experimental animal models [1, 4, 5, 15–18, 20–25] or clinical human studies enrolling cancer patients [6, 19]. However, to the best of our knowledge, this is the first report on using a traditional folk medicine to reverse docetaxel-induced myelotoxicity and on the myelo-protective effect of *Phragmitis rhizoma*.

In this study, we showed that the aqueous extract of *Phragmitis rhizoma* (EPR) ameliorated docetaxel-induced myelosuppression in both in vitro and in vivo experimental models. In summary, EPR efficiently reduced myelotoxicity induced by anticancer agents that have different mechanisms of action (docetaxel, CPT11 and doxorubicin; Fig. 2 and Additional file 1: Figure S1). In our mouse model, repeated injection of docetaxel remarkably decreased the WBC (38%), neutrophil (38%) and lymphocyte (46%) counts in the blood. RBC (15%) were more resistant to docetaxel toxicity than that of the WBC, which may be due to their longer lifespan compared to WBC in the circulating blood [25]. Unexpectedly, the platelet count was increased by docetaxel which was consistent with the previous clinical study reporting that co-administration of docetaxel could spare platelet counts in cancer patients who received carboplatin by elevating circulation TPO concentration in blood [26]. EPR could slightly attenuate this docetaxel-induced platelet increase. Thus, the EPR treatment had the following benefits: blood cell counts rebounded (Table 3), microenvironmental niches in the bone marrow improved, and the structural integrity of the thymus was preserved from docetaxel toxicity (Fig. 3).

In our animal model, IL3 expression was significantly suppressed in splenic tissues following exposure to docetaxel. IL6 expression did not change but SCF and GM-CSF were slightly increased by the docetaxel treatment. Elevated expression of immune-stimulating cytokines has also been observed in other different myelosuppressed animal models. For example Liu et al. reported that IL1β, IL3 and IL6 expression was increased in spleens, and IL6, SCF and GM-CSF expression was increased in bone marrow stromal cells in combined radiotherapy/cyclophosphamide/chloramphenicol-induced myelosuppressed mice [4]. In our docetaxel model, it was shown that EPR relieved the suppressed IL3 expression and promoted the expression of immune-stimulating cytokines like IL6, SCF and GM-CSF in splenic tissues (Fig. 4). Furthermore, IL3, IL6, IFNγ and TNFα were induced by EPR in normal splenocytes (Fig. 6) whereas IL1β and IFNγ were induced in bone marrow cells (Table 4). The different patterns of cytokine expression response to EPR may be partially due to different cell populations residing in two immune organs.

IL3 is known to potentially support the proliferation and expansion of early myeloid progenitors leading to bone marrow reconstitution, aplastic anemia and hematopoietic recovery following chemotherapy [27]. GM-CSF stimulates hematopoietic stem cells to produce granulocytes and macrophage myeloid cells [5]. Except for GM-CSF and IL3, other cytokines and SCF are known to have little or no effect on the proliferation of hematopoietic progenitor cells when acting alone but enhance the response to other HGFs [28]. IL1β, an isoform of IL1, is known to induce a variety of HGFs including GM-CSF. IL6 is known to stimulate hematopoietic stem cells, granulocyte-macrophage progenitor cells and erythroid progenitor cells, and can accelerate hematopoietic recovery [29]. A synergistic myelostimulating effect was observed between IL1β and IL6 in cyclophosphamide-induced myelosuppressed mice which was not recovered by single regime [28]. Administration of IL1 with IL6 reconstitutes hematopoietic functions in chemotherapy/radiation-induced myelosuppressed mice [30]. Enhancement of IFNγ expression may have a positive role in immune suppressed cancer patients by providing them protective immunity against infections [5]. TNFα is known to suppress the growth of erythroid and myeloid progenitors resulting in the inhibition of erythropoiesis. However, TNFα can mediate the maturation of a granulocyte-macrophage lineage and may protect myelosuppressed cancer patients from bacterial infections by increasing bacteriocidal phagocytic cytotoxicity [5, 31]. Therefore, EPR may exert its myelo-protective effect by inducing the expression of diverse hematopoietic cytokines and by their combinatorial effects. In addition, EPR and its major component *p*-hydroxycinnamic acid could ameliorated docetaxel-induced myelosuppression in vitro (Fig. 2) and promote the proliferation of splenocytes and thymocytes (Fig. 5). However, *p*-hydroxycinnamic acid did not remarkably modulate immune-stimulating cytokines in splenocytes as much as EPR (data not shown). Taken together, *p*-hydroxycinnamic acid partially contributes to the protective activity of EPR against docetaxel myelotoxicity. Myelo-protective effect of *p*-hydroxycinnamic acid should be confirmed in in vivo animal model in our future study. In the present study we cannot rule out the fact that active substances other than *p*-hydroxycinnamic acid or combination among them may also contribute to myelo-protective potential of EPR. The identity of other component(s) should be further elucidated to fully understand the mechanism of the EPR.

Prediction of potential toxicity of a candidate at the early drug developmental stage is critical to minimize the risk of failure. Especially, drug-induced liver injury is the most frequent cause of post-marketing warnings and withdrawals [32]. In this study, we also estimated the toxicity of the EPR in 2 normal human fibroblasts (IMR-90 and HFFn) and 1 normal endothelial (HUVEC) cells. In all the tested cells, the EPR did not show any remarkable toxicities within 0–200 μg/ml ranges of the EPR. Furthermore, we predicted the hepatotoxicity of the EPR using multi-parametric hepatotoxicity tests including cell viability, apoptotic caspase 3/7 activation, intracellular GSH contents and LDH leakage in HepaRG. Although the HepaRG hepatic cell line was constructed from a human hepatocellular carcinoma [33], they still contain a variety of enzyme activities seen in primary human hepatocytes, such as cytochrome P450s, P-gp membrane transporters and phase II metabolizing enzymes that are responsible for normal liver function [32, 34]. No remarkable hepatotoxicity in the HepaRG was observed even when the cells were exposed to up to 500 μg/ml EPR. Overall, EPR is a safe and potential candidate for novel drug development to treat chemotherapy-induced myelosuppression in cancer patients based on the present efficacy and toxicity studies.

## Conclusions

*Phragmitis rhizoma* extract had a protective effect against docetaxel-induced myelotoxicity by in part preserving normal cellularity and organ structure like bone marrow and thymus, enhancing hematopoietic stimulant cytokines and promotion of immune cell proliferation without any remarkable toxicities in normal cells. Therefore, *Phragmitis rhizoma* extract can be considered as a potential candidate for further developing novel myelo-protective agents to manage cancer patients. Further studies, however, should be done to elucidate molecular targets and active constituents of EPR to better understand the underlying mechanism of action.

## Additional file

Additional file 1: Figure S1. In vitro myelo-protective effects of *Phragmitis rhizoma* extract. (PPTX 43 kb)

## Abbreviations

BMNC: bone marrow nucleated cell
CBC: Complete blood count
CFU: colony forming unit
CMC: carboxymethylcellulose
EDTA: ethylenediaminetetraacetic acid
ELISA: enzyme-linked immunosorbent assay
EPR: aqueous extract of *Phragmitis rhizoma*
FBS: fetal bovine serum
G-CSF: granulocyte-colony stimulating factor
GM-CSF: granulocyte/macrophage-colony stimulating factor
GSH: glutathione
H&E: hematoxylin-eosin
HBSS: Hank’s balanced salt solution
HFFn: neonatal foreskin fibroblast
HGF: hematopoietic growth factors
HPLC: high-performance liquid chromatography
HUVEC: human umbilical vein endothelial cells
IFNγ: interferon-gamma
IL3: interleukin-3
IL6: interleukin-6
LDH: lactate dehydrogenase
PBS: phosphate buffered-saline
p-HCA: *p*-hydroxycinnamic acid
qPCR: quantitative real-time polymerase chain reaction
RBC: red blood cells
SCF: stem cell factor
TC_50_: toxic concentrations that decrease the response by 50%
TCM: traditional Chinese medicine
TKM: traditional Korean medicine
TNFα: tumor necrosis factor-alpha
UHPLC: ultra-high performance liquid chromatographic; WBC, white blood cells
