# Supplementary material for: Aqueous extract of Phragmitis rhizoma ameliorates myelotoxicity of docetaxel in vitro and in vivo
Source: BMC Complement Altern Med. 2017 Aug 9;17:393. doi: 10.1186/s12906-017-1890-1 (PMC5549314; doi:10.1186/s12906-017-1890-1)
Supplement: Additional file 1: Figure S1. — In vitro myelo-protective effects of Phragmitis rhizoma extract. (PPTX 43 kb) [file 12906_2017_1890_MOESM1_ESM.pptx]

## Slide 1
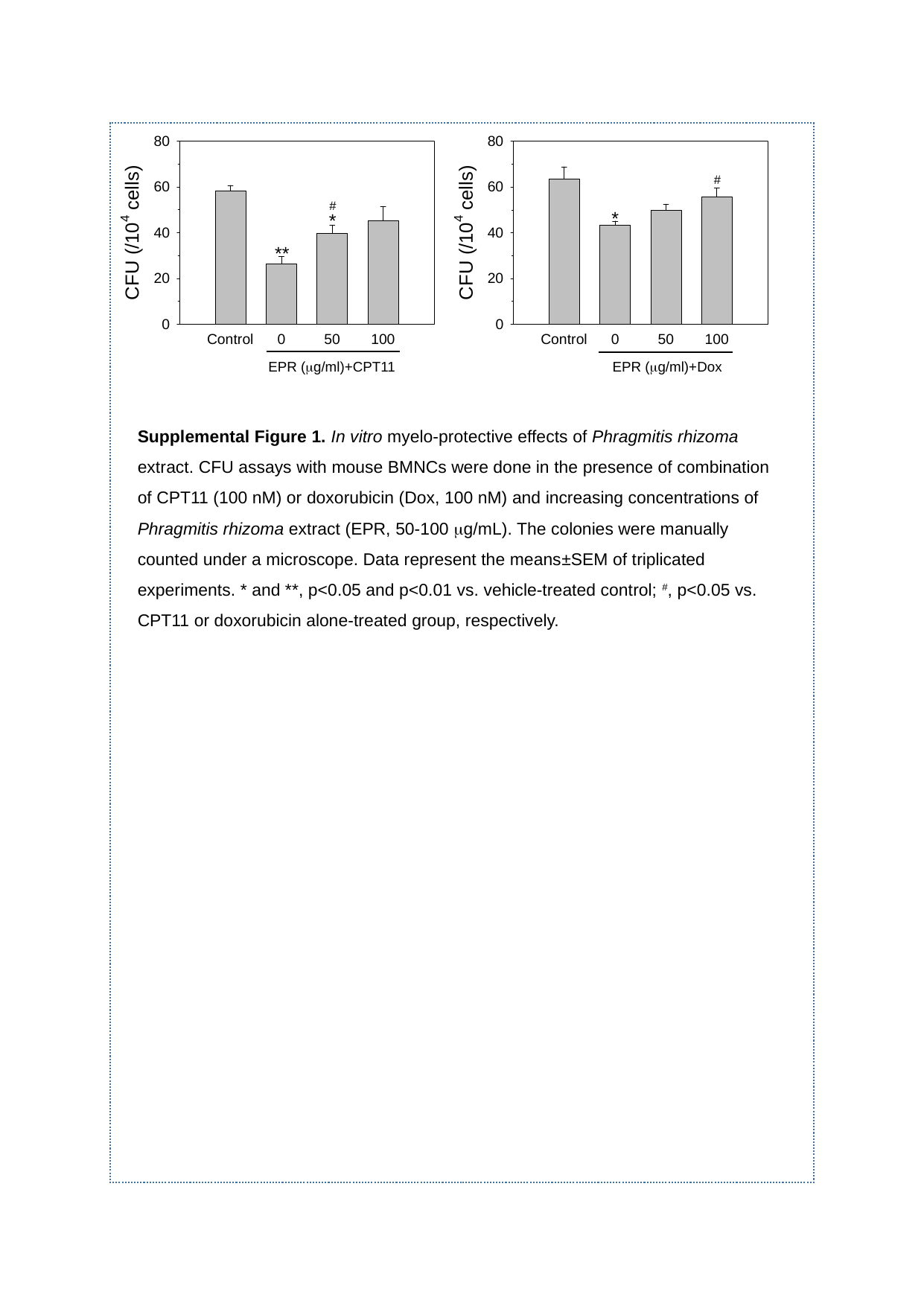

#
#
*
*
**
EPR (mg/ml)+CPT11
EPR (mg/ml)+Dox
Supplemental Figure 1. In vitro myelo-protective effects of Phragmitis rhizoma extract. CFU assays with mouse BMNCs were done in the presence of combination of CPT11 (100 nM) or doxorubicin (Dox, 100 nM) and increasing concentrations of Phragmitis rhizoma extract (EPR, 50-100 mg/mL). The colonies were manually counted under a microscope. Data represent the means±SEM of triplicated experiments. * and **, p<0.05 and p<0.01 vs. vehicle-treated control; #, p<0.05 vs. CPT11 or doxorubicin alone-treated group, respectively.
